# Polycondensed Peptide Carriers Modified with Cyclic RGD Ligand for Targeted Suicide Gene Delivery to Uterine Fibroid Cells

**DOI:** 10.3390/ijms23031164

**Published:** 2022-01-21

**Authors:** Anna Egorova, Sofia Shtykalova, Marianna Maretina, Alexander Selutin, Natalia Shved, Dmitriy Deviatkin, Sergey Selkov, Vladislav Baranov, Anton Kiselev

**Affiliations:** 1 Department of Genomic Medicine, D.O. Ott Research Institute of Obstetrics, Gynecology and Reproductology, Mendeleevskaya Line 3, 199034 Saint Petersburg, Russia; egorova_anna@yahoo.com (A.E.); sofia.shtykalova@gmail.com (S.S.); marianna0204@gmail.com (M.M.); natashved@mail.ru (N.S.); dimi02121@gmail.com (D.D.); baranov@vb2475.spb.edu (V.B.); 2 Department of Immunology and Intercellular Interactions, D.O. Ott Research Institute of Obstetrics, Gynecology and Reproductology, Mendeleevskaya Line 3, 199034 Saint Petersburg, Russia; a_selutin@yahoo.com (A.S.); selkovsa@mail.ru (S.S.)

**Keywords:** DNA delivery, peptide-based carriers, gene therapy, thymidine kinase, uterine fibroids, integrins, polycondensation

## Abstract

Suicide gene therapy was suggested as a possible strategy for the treatment of uterine fibroids (UFs), which are the most common benign tumors inwomen of reproductive age. For successful suicide gene therapy, DNAtherapeutics should be specifically delivered to UF cells. Peptide carriers are promising non-viral gene delivery systems that can be easily modified with ligands and other biomolecules to overcome DNA transfer barriers. Here we designed polycondensed peptide carriers modified with a cyclic RGD moiety for targeted DNA delivery to UF cells. Molecular weights of the resultant polymers were determined, and inclusion of the ligand was confirmed by MALDI-TOF. The physicochemical properties of the polyplexes, as well as cellular DNA transport, toxicity, and transfection efficiency were studied, and the specificity of αvβ3 integrin-expressing cell transfection was proved. The modification with the ligand resulted in a three-fold increase of transfection efficiency. Modeling of the suicide gene therapy by transferring the HSV-TK suicide gene to primary cells obtained from myomatous nodes of uterine leiomyoma patients was carried out. We observed up to a 2.3-fold decrease in proliferative activity after ganciclovir treatment of the transfected cells. Pro- and anti-apoptotic gene expression analysis confirmed our findings that the developed polyplexes stimulate UF cell death in a suicide-specific manner.

## 1. Introduction

Gene therapy is a treatment by delivery of nucleic acid constructs into cells with the aim of repairing, adding, or removing a genetic sequence, or targeting genetic information processes [1]. Gene therapy can be used to treat a wide range of inherited and acquired diseases. According to the latest data from The Journal of Gene Medicine, most gene therapy clinical trials focus on the treatment of both malignant and benign tumors (http://www.abedia.com/wiley; accessed on 1 September 2021).

The most common benign tumors in women of reproductive age are uterine fibroids (UFs). UFs can cause profuse uterine bleeding, pelvic pain, complications inpregnancy, and infertility, and they arethe most common cause of myomectomy [2,3]. There are three major types of UF—submucosal, subserosal, and intramural fibroids [4]. The latter grow within the muscular uterine wall and imply certain difficulties and increased traumatism of the healthy myometrium during myomectomy, which makes this approach undesirable for treatment of intramural fibroids in comparison with hormonal therapy [5]. In fact, in the case of UFs with an intramural location in infertile women, the surgical treatment is controversial as there is no evidence that a myomectomy increases spontaneous or IVF-assisted fertility [4,6]. The fibroids are easily accessible by various endoscopic methods that make this disease a promising target for in situ gene therapy [7]. Delivery of apoptosis-inducing genes can be an effective approach to UF gene therapy. Most studies on suicide gene therapy use the delivery of the herpes simplex virus thymidine kinase (HSV-TK) gene followed by treatment with prodrugs, such as acyclovir or ganciclovir (GCV) [8]. A significant advantage of this approach is the so-called “bystander effect”, the phenomenon based on the fact that phosphorylated GCV enters neighboring non-transfected cells through intercellular contacts widely spread in UF cells compared to normal myometrium; this in fact increases the efficiency of therapy without negative effects on healthy tissues [9,10]. Thus, the proposed gene therapy approach may be beneficial for UF treatment, especially for fibroids with an intramural location.

One of the main reasons for the limited widespread application of gene therapy is the lack of simultaneously effective, safe, and inexpensive methods for nucleic acid (NA) delivery. It is known that in most cases unprotected NAs are not able to overcome extracellular and intracellular barriers independently and easily degrade [11]. To date, the adenovirus (Ad) vectors are the most studied as delivery vehicles for UF gene therapy. The various modifications of Ad vectors are being developed to increase the efficiency and specificity of gene delivery into UF cells in vitro and in vivo [12,13,14,15]. However, the use of Ad vectors for systemic gene delivery is associated with the risk of the virus capsid proteins’ interaction with blood components whichcan cause a systemic inflammatory response [16].

Non-viral vectors are being actively developed as an alternate delivery method in order to improve safety, reduce the difficulties in the production and storage of gene therapeutics, and also lower their cost. In terms of efficiency, they are still inferior to viral vectors, but these compounds are actively being developed in order to demonstrate their promise as successful gene delivery vectors for clinical use. The first application of the non-viral approach for HSV-TK gene delivery into UF cells was demonstrated earlier by Niu and colleagues using calcium phosphate-facilitated transfection. The authors emphasized the advantage of non-viral vector application associated with the lack of immune response to pDNA delivery and the susceptibility of UF cells to non-viral gene transfer [17]. 

Today, cationic lipids and polymers are the most promising non-viral gene delivery systems. Cationic lipids require extensive formulation work to optimize combinations and concentrations of liposomal components, whereas cationic polymers tend to complex with nucleic acids into smaller and more uniform nanoparticles. Cationic peptide carriers based on the cell-penetrating peptides (CPP) have additional advantages, such as biodegradability and better interaction with cellular membranes, enhancing transfection efficiency [18,19,20,21]. Arginine-rich CPPs form stable complexes with nucleic acids and promote their intracellular uptake due to hydrogen bond formation with various functional groups of cell surface glycosaminoglycans [22,23]. The structure of peptide-based carriers can be easily modified by the inclusion of different functional amino acids. For example, the inclusion of histidine provides the release of complexes into the cytoplasm after endosome destruction due to the “proton sponge” effect [24,25], whereas the inclusion of terminal cysteines enables the formation of higher molecular weight polypeptide-like polymers cross-linked by intermolecular disulfide bonds [26]. In the cytoplasm, the disulfide bonds are degraded by glutathione with the subsequent NA release, while the carrier toxicity is decreased to the level of low-molecular weight peptides [27]. There are two approaches to the formation of cross-linked polypeptide-like polymers: matrix polymerization, where interpeptide disulfide bonds are formed during NA complexation, and oxidative polycondensation, using dimethyl sulfoxide (DMSO) as a catalytic agent [28,29]. A number of studies have shown an advantage in transfection efficiency of the polycondensed peptide carriers compared to the matrix-polymerized carriers [26,29,30]. Recently, we observed a significant decrease in the survival of GCV-treated primary UF cells after delivery of the HSV-TK gene mediated by the polycondensed peptide carrier, polyR [31].

For successful gene therapy implementation, the NA complexes have to enter the cell specifically. In tumor gene therapy, the widespread approach is to modify carriers with the RGD motif (arginine–glycine–aspartic acid) for binding cell surface αvβ3 integrins. This type of integrin is overexpressed on the surface of tumor cells compared to normal tissues [32]. Importantly, αvβ3 integrins are widely expressed in UF cells but not in normal myometrium, which makes them promising for targeted UF gene therapy [33]. Previously, an increase in efficiency and specificity of UF cell transduction after Ad vector modification with the cyclic RGD (arginine–glycine–aspartic acid) ligand has been demonstrated [12,13,14]. In addition, we showed recently that combining the matrix-polymerized arginine-rich peptide carriers with the iRGD-modified peptides resulted in an increase in cell transfection efficiency and successful suicide gene therapy of UF primary cells [34].

Here, we present the development and detailed characterization of polycondensed peptide carriers modified with a cyclic RGD moiety during the polycondensation process. Molecular weights of the resultant polymers and inclusion of the ligand were determined by MALDI-TOF mass spectrometry. The physicochemical properties of the obtained peptide/DNA complexes (the size and zeta-potential of the nanoparticles, the efficiency of DNA complex formation, etc.), as well as the quantitative assessment of the complexes’ transport into cells, toxicity, transfection efficiency, and specificity were studied in vitro. Modeling of the suicide gene therapy by the HSV-TK gene transfer into primary cells obtained from myomatous nodes of patients with UFs, followed by an assessment of the proliferative activity, apoptosis of the UF cells, along with apoptosis marker expression, was carried out.

## 2. Results and Discussion

### 2.1. Carrier Design and Molecular Weight Characterization

In this work, we obtained the R6p-cRGD carrier via oxidative polycondensation of arginine–histidine–cysteine-rich peptide R6 monomers with the inclusion of cyclic RGD-ligand in the reaction mixture (Table 1). The combination of arginine, histidine, and cysteine in the composition of the peptide-based gene delivery systems was proven to facilitate the effective entry of NA complexes into the cells and provide endosome disruption [35,36,37]. The cysteine thiol group in the composition of the cyclic RGDligand was protected by a 3-nitro-2-pyridinesulfenyl group (Npys), which acts as an activating group for disulfide bond formation, as it is displaced by the free thiol during oxidative polycondensation of R6 monomers [38]. Previously, we have shown that the peptide-based polyplexes require not less than 50% modification by the RGDligand to achievespecific gene delivery into αvβ3 integrin-expressing cells [37]. Due to this fact, we included a cyclic RGD moiety in the reaction mixture at the rate of one ligand molecule per two R6 monomers.

The molecular weight of the R6p and R6p-cRGD carriers formed by oxidative polycondensation was determined with MALDI-TOF mass spectrometry (Tables S1 and S2). It was found that the molecular weight of formed R6p polypeptides varied within 2.87–27.42 kDa, which corresponds to polymers with a degree polymerization of monomers from 2 to 19. MALDI-TOF analysis of R6p-cRGD also revealed a mixture of oligomers corresponding to 1–18 peptide monomers modified with the cRGD ligand. It should be noted that the absence or partial cRGD modification was also observed (Table S2). Nevertheless, it can be concluded that the addition of the cRGD ligand bearing a Npys-protected thiol group during polycondensation results in successful carrier modification. Moreover, despite the fact that cRGD is essentially a chain breaker during polycondensation, we did not observe a significant decrease in the degree of polymerization of the R6p-cRGD carrier.

### 2.2. DNA-Binding and DNA Protection Properties of the Carrier

**Figure 1:**
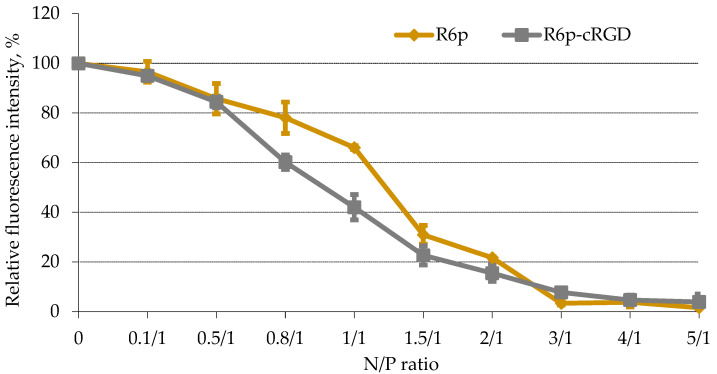
EtBr displacement assay of DNA complexes with R6p and R6p-cRGD carriers. Values are the mean ± SD of the mean of triplicates.

The degree of DNA binding with the polycondensed carriers was assessed by ethidium bromide displacement assay (Figure 1). An increase of the carriers’ concentration gradually resulted in an increase in the density of complexes. The EtBr fluorescence intensity in peptide/DNA complexes sharply decreased at anN/P ratio of three(up to 3–8%) compared to that of naked DNA (100%).

**Figure 2:**
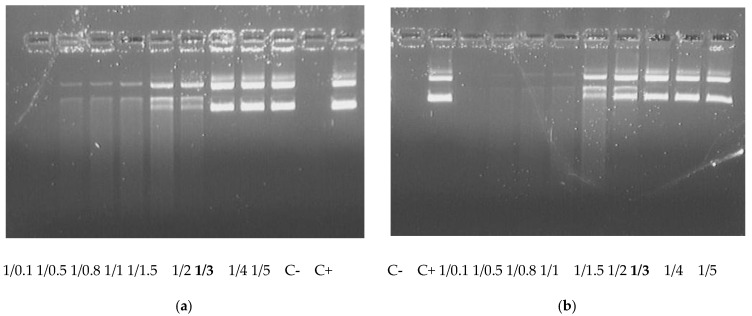
DNase I protection assay of DNAcomplexes formed with R6p (**a**) and R6p-cRGD (**b**) carriers. Charge ratio (CR) in **bold** indicates full DNA protection. C– = ‘naked’ plasmid DNA treated with DNase I; C+ = untreated plasmid DNA.

The DNA-binding capacity of carriers directly influences nuclease protection properties [39]. DNA integrity in the complexes was estimated using a DNase I protection assay. The studied polycondensed carriers were able to protect DNA from nuclease degradation at the same N/P ratio of three which corresponds to full DNA condensation (Figure 2).

Thus, the obtained results showed no effect of the ligand inclusion inthe R6p-cRGD carrier composition on its DNA-binding and protection abilities. This result may have a positive effect on the specificity of DNA delivery because no interference between DNA-binding and receptor-binding properties could be expected.

### 2.3. Size and ʐ-Potential of the Carrier/DNA Complexes

The size of peptide/DNA complexes is one of the key parameters affecting the mechanism of cell penetration [40]. For example, 100–200 nm-size complexes enter the cell via clathrin-mediated endocytosis, while larger ones penetrate into the cell by macropinocytosis [41]. Caveolin-mediated endocytosis, or potocytosis, may be used when the particle size does not exceed 100 nm [42]. Previous studies of non-viral carrier/DNA complexes revealed that particles of 70–200 nm have the longest duration of bloodstream circulation and the highest efficiency of tumor penetration [43]. We determined the size and ʐ-potential of carrier/DNA complexes at N/P ratios 8/1 and 12/1, which provide full DNA binding/protection and should be colloidally stable. It was found that the size of the studied peptide/DNA complexes varied in the range of 100–200 nm (Table 2), and thus in the optimal range for cell membrane penetration via clathrin-mediated endocytosis. Importantly, cRGD ligand-modified nanoparticles were shown to internalize through the clathrin-mediated pathway [44].

The surface charge density of the carrier/DNA complex plays an important role in gene delivery efficiency [45]. To begin with, the positive charge of the carrier is substantial for DNA condensation, and the positive surface charge of the formed complex may increase cellular uptake due to electrostatic interaction with the negatively charged cell membrane [46]. The studied peptide/DNA polyplexes were found to be positively charged; their ʐ-potential varied within a range from +30 to +36 mV (Table 2). This may contribute to increased cellular uptake and, as a result, to enhanced gene expression.

### 2.4. DTT and DS Treatment of the Complexes

**Figure 3:**
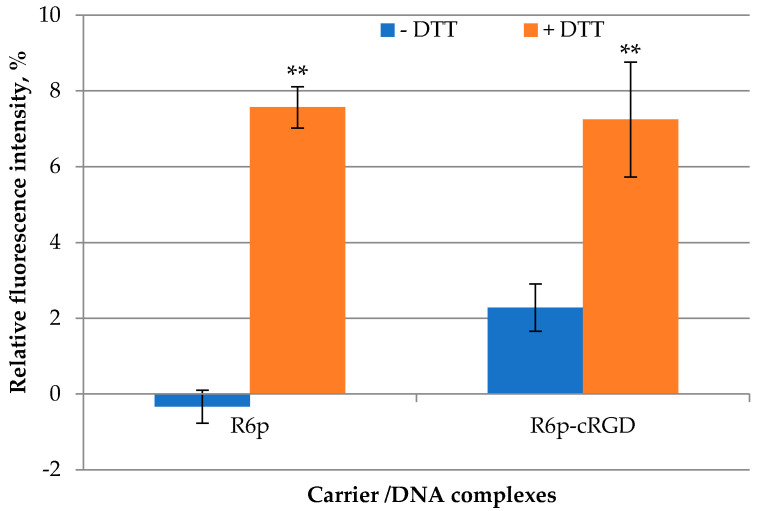
DNA release after DTT treatment of DNAcomplexes with R6p and R6p-cRGD carriers formed at anN/P ratio 8/1. Values are the mean ± SEM of the mean of triplicates. ** *p* < 0.01 compared to untreated complexes.

**Figure 4:**
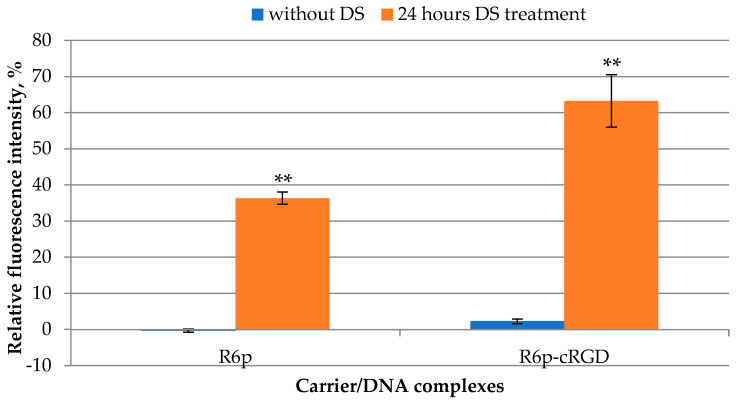
Relaxation of DNAcomplexes with R6p and R6p-cRGD carriers (N/P ratio 8/1) after 24 h of DS treatment in three-fold charge excess. Values are the mean ± SEM of the mean of triplicates. ** *p* < 0.01 compared to untreated complexes.

DNA release is an essential step for the manifestation of its bioactivity [47]. R6p and R6p-cRGD carriers contain disulfide bonds designed to facilitate DNA release into the cytoplasm. In order to determine the rate of the DNA release after disulfide bond reduction, we used dithiothreitol (DTT) at a concentration of 200 mM to mimic a cytoplasmic glutathione interaction [48]. Before DTT treatment, we ensured complete DNA condensation by the tested carriers at a charge ratio of 8/1. DTT treatment for 1 h resulted in a 2–7-fold increase in fluorescence intensity, which corresponds to DNA release from the polyplexes (Figure 3). However, the relative level of fluorescence after incubation with DTT compared to free DNA (100%) was not very high, which indicates that electrostatic interaction is more important for the stability of complexes between DNA and arginine-rich carriers [34]. Additional data on DNA release were obtained after treatment of the complexes with negatively charged glycosaminoglycan in three-fold charge excess (Figure 4).

Glycosaminoglycans (GAGs), such as heparan sulfate (HS), chondroitin sulfate (CS), and dextran sulfate (DS), influence several aspects of peptide-mediated DNA transfer. It has been shown that GAGs may play a protective role against cytotoxicity associated with polycations [49]. On the other hand, they are capable of disrupting peptide/DNA complex integrity in the extracellular environment and significantly inhibit gene transfection [50,51]. After 24 h of DS treatment, an increase in the fluorescence intensity was detected, due to the relaxation of these complexes and a decrease in their density (Figure 4). For R6p and R6p-cRGD polyplexes, the fluorescence increased from 0.3% to 36.3% and from 2.3% to 63.3%, respectively. Notably, the cRGD-modified carrier released DNA more efficiently than the R6p carrier (Figure 4). 

A delicate balance holds between DNA condensation and release. Too weak DNA binding might be insufficient to protect DNA against extra- and intracellular nuclease degradation, whereas too tight DNA binding might slow down or prevent DNA release from the carrier and hinder gene transcription [52]. A better understanding of nucleic acid binding mechanisms would be beneficial in designing more efficient gene delivery systems.

### 2.5. Cytotoxicity of Peptide/DNA Complexes

**Figure 5:**
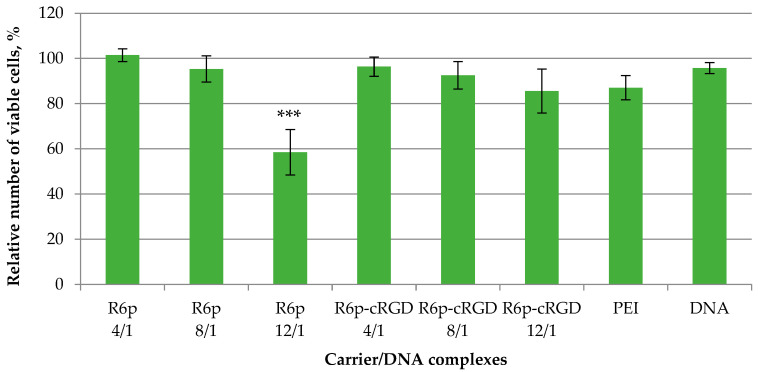
Cytotoxicity after transfection of DNA complexes formed with RGD1-R6, RGD0-R6, and R6 carriers at charge ratios 4/1, 8/1, and 12/1. Values are the mean ± SD of the mean of triplicates. *** *p* < 0.001 compared to intact cells.

Gene transfection usually correlates with the perturbation of cellular membranes, which may elicit unwanted cytotoxicity [53,54]. Moreover, the relatively high net positive charge density of the carrier/DNA complex (>30 mV) also might induce cellular toxicity [55]. It is important to develop gene delivery carriers that have reduced toxicity without a decrease in transfection efficiency. One strategy is the incorporation of biodegradable linkers in the carrier composition [56,57]. The inclusion of the disulfide interpeptide bonds in R6p and R6p-cRGD polymers resulted in their cytoplasmic reduction, which was confirmed by DNA release after DTT treatment (Figure 4). A decrease in carrier molecular weight and, as a result, the reduction of positive charge density could decrease cytotoxicity. The cytotoxicity of R6p/DNA and R6p-cRGD/DNA complexes was determined in αvβ3-positive PANC-1 cells (Figure 5). Naked DNA was used as a negative control, and PEI/DNA complexes at an N/P ratio of 8/1 were used as a positive control. For the R6p/DNA complexes, cell viability exhibited a slightly decreasing trend with the increase of the N/P ratio and was significantly lower than that of intact cells and naked DNA. In the case of R6p/DNA complexes at anN/P of 12/1, the numberof living cells was reduced to 58% and was lower compared to PEI/DNA complexes. However, the R6p-cRGD/DNA complexes had relatively low cytotoxicity in PANC-1 cells at all tested N/P ratios; the cell viability after the transfection was comparable with that of naked DNA.

### 2.6. Cellular Uptake

**Figure 6:**
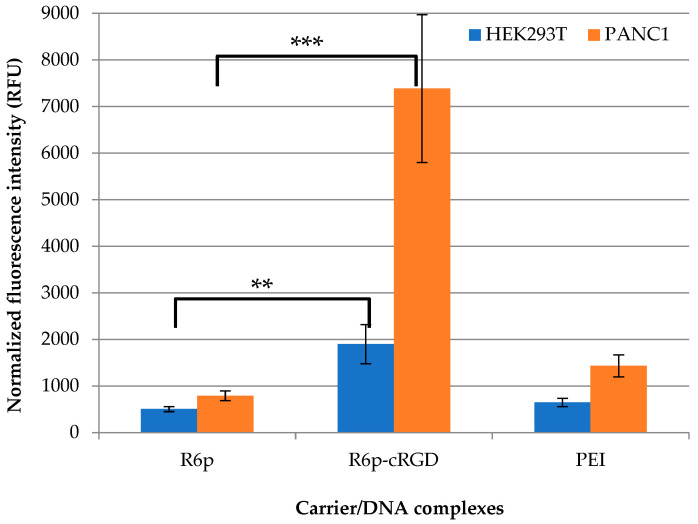
Normalized fluorescence intensity of PANC-1 and 293T cells after uptake of R6p/DNA and R6p-cRGD/DNA complexes at 8/1 charge ratios labeled with YOYO-1. ** *p* < 0.01, *** *p* < 0.001, when compared with R6p/DNA polyplexes.

The intracellular uptake of DNA condensed by R6p or R6p-cRGD was determined by flow cytometry (Figure 6). To prove targeted DNA delivery with the R6p-cRGD carrier we used αvβ3-negative 293T cells and αvβ3-positive PANC-1 cells, which express a different level of αvβ3integrins on their surface [37]. The normalized fluorescence intensity of YOYO-1-labeled DNA taken up by cells 2 h after transfection is demonstrated in Figure 6. The complexes were studied at an 8/1 charge ratio because they do not exhibit toxicity at this N/P ratio, and no decrease in cellular uptake can be expected due to possible negative effects on the cell membrane (Figure 5). In PANC-1 cells, R6p-cRGD/DNA complexes showed higher intracellular uptake efficiency, 9.3times more efficient than R6p/DNA complexes. The data obtained are consistent with the other studies that proved an increase in the penetrating ability of the polyplexes modified with the αvβ3integrin ligand [58]. For 293T cells, differences in the cell penetration efficiency of ligand-modified and unmodified polyplexes were also found. However, R6p-cRGD/DNA polyplexes entered the cell only 3.7 times more efficiently than R6p/DNA complexes. Thus, we observed a direct correlationbetween the intracellular uptake efficiency of complexes and the presence of αvβ3 integrins on cell surfaces. It should be noted, that the cellular uptake efficiency depends not only on the presence of the ligand part in the carrier but also on polyplex size and other physicochemical properties [42]. Importantly, the size of R6p/DNA and R6p-cRGD/DNA complexes lies in the range of 100–200 nm (Table 2) and is optimal for clathrin-mediated endocytosis. Thus, it can be concluded that the polyplexes likely do not differ in terms of penetration, which further confirms the specificity of R6p-cRGD/DNA polyplexes uptake by receptor-mediated endocytosis via αvβ3 integrins.

### 2.7. Transfection Studies

**Figure 7:**
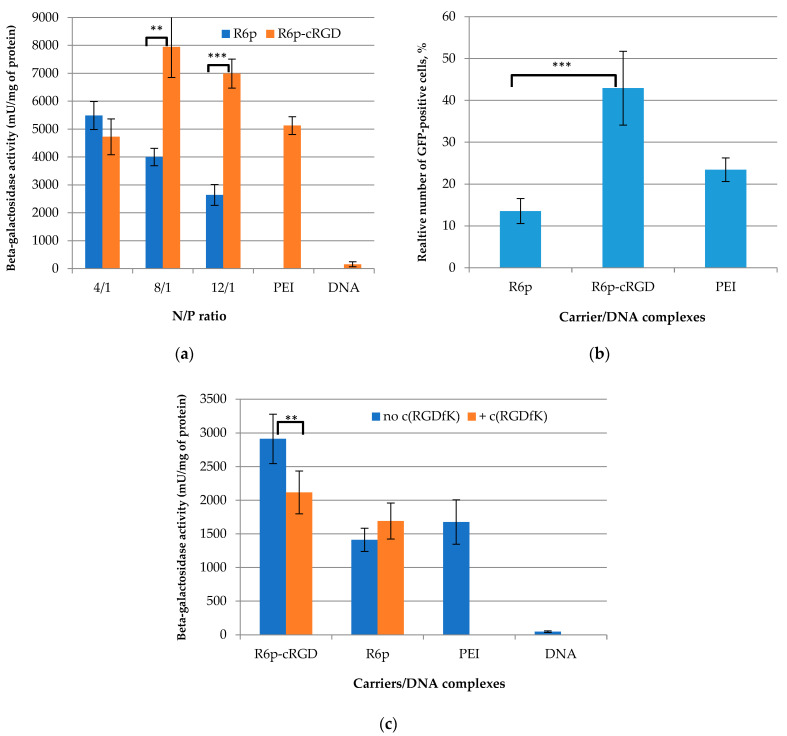
Transfection efficacy evaluation in the PANC-1 cells: (**a**) the cells were transfected with R6p/DNA and R6p-cRGD/DNA complexes at charge ratios of 4/1, 8/1, and 12/1, ‘naked’ plasmid pCMV-lacZ alone, and PEI/DNA complexes; (**b**) the cells were transfected with complexes of the pEXPR-IBA5-eGFP plasmid with R6p and R6p-cRGD carriers at a charge ratio of 8/1 withPEI/DNA polyplexes as a control; (**c**) the cells were transfected in the presence of the free cyclo(RGDfK) ligand with R6p/DNA and R6p-cRGD/DNA complexes at a charge ratio of 8/1, with thepCMV-lacZ plasmid alone and PEI/DNA polyplexes used as controls. *lacZ* gene expression was calculated as milliunits (mU) of beta-galactosidase per milligram of total cell extract protein. GFP gene expression was presented as a percentage of GFP-positive cells determined by flow cytometry assay. Values are the mean ± SD of the mean of triplicates. ** *p* < 0.01, *** *p* < 0.001, when compared with R6p/DNA polyplexes (**a**,**b**) and after free cyclo(RGDfK) ligand addition in transfection medium (**c**).

In order to determine the transfection efficiency of R6p/DNA and R6p-cRGD/DNA complexes in αvβ3-positive PANC-1 cells, pCMV-lacZ was used as a reporter gene plasmid. PANC-1 cells were treated with the polyplexes at N/P ratios of 4/1, 8/1, and 12/1, with pCMV-lacZ only as a negative control and PEI/DNA complexes as a positive one (Figure 7a). At a 4/1 charge ratio, a significant difference in the transfection efficacy of ligand-modified and non-modified polyplexes was not found. As we showed previously at this charge ratio, iRGD-modified polyplexes could not provide ligand-mediated cellular penetration and subsequently could not result in enhanced transfection efficacy [37]. Beta-galactosidase activity after cell transfection with R6p-cRGD/DNA polyplexes at 8/1 and 12/1 N/P ratios was 2–2.5 times higher than that with R6p/DNA complexes; moreover, in most cases the transfection efficacy of ligand-modified polyplexes exceeded that of PEI/DNA complexes. These results demonstrate an increased transfection activity of R6p-cRGD/DNA polyplexes that was additionally proved using pEXPR-IBA5-eGFP plasmid transfer. In this instance, the transfection efficiency of PANC-1 cells mediated by the complexes was evaluated by flow cytometry analysis and estimated as a percentage (%) of transfected cells (Figure 7b). The R6p-cRGD/DNA transfection resulted in 43% of GFP-positive PANC-1 cells compared with 13.5% of GFP-positive cells after using DNA R6p/DNA complexes. Moreover, the efficacy of R6p-cRGD/DNA complexes was higher than that of PEI polyplexes (24%). The result demonstrated the high efficiency of R6p-cRGD polypeptides for DNA delivery in αvβ3-positive PANC-1 cells, and, thus, indirectly confirmed the specificity of gene transfer by the cRGD-modified carrier.

In order to further prove the selectivity of the R6p-cRGD-mediated transfection of αvβ3-positive cells, PANC-1 cells were pre-treated with free cyclo(RGDfK) peptide ligand. To perform competitive studies PANC-1 cells were transfected with R6p/DNA and R6p-cRGD/DNA complexes at an N/P ratio of 8/1 in the presence of a 10-fold excess of cyclo(RGDfK) (Figure 7c). The cell pre-treatment resulted in a 27% decrease in the efficacy of the R6p-cRGD/DNA polyplexes to the level of R6p/DNA complexes. However, the transfection efficiency after cell treatment with R6p/DNA polyplexes was not changed in the presence of the cyclo(RGDfK) ligand. Thus, we demonstrated the direct involvement of the cRGD ligand in the complexes’ transfection via αvβ3 integrin binding. 

To sum up, the obtained results proved that the cRGD-modified peptide carriers are favorable for transfection of αvβ3-positive cell lines, due to the specific recognition of the ligand-modified polyplexes by these cells that is beneficial for cellular uptake and the transfection process. So, the polycondensed cRGD-modified carrier can be applied as a targeted gene delivery vehicle for gene therapy purposes. Previously, it was shown that αvβ3 integrin targeting increases UF cells transfection efficacy compared to normal myometrial cells, indicating that an RGD moiety can selectively target uterine fibroids [13].

### 2.8. Assessment of Suicide Gene Therapy Effect in GSV-Treated Uterine Fibroid Cells after R6p-cRGD/pPTK1 Polyplex Transfection 

There are no conservative and sufficiently safe approaches to UF therapy for women who want to keep their fertile potential [59]. The HSV-TK/GCV suicide gene therapy seems to be one of the most promising therapeutic strategies for UF treatment as a localized and organ-preserving method. The strategy has been actively developed for UF therapy and its successful application has been demonstrated in in vitro and in vivo studies [7]. The experimentally observed “bystander effect” arising from a large number of gap-junctions in uterine leiomyoma cells greatly enhanced the efficiency of this gene therapy approach [10,17]. It seems important that 48% of human UF cell death occurred when only 5% of the cells were transfected with the HSV-TK gene [10]. In addition, the reduced level of these junctions in normal myometrium compared to UFs will allow safety in suicide gene therapy for surrounding tissues. Thus, the studied approach could be a basis for future clinical trials of uterine leiomyoma. 

UF cells overexpress αvβ3 integrins on their surface; our previous data demonstrated that up to 73% of UF cells can contain these receptors [34]. Importantly, when both viral and non-viral vehicles were modified with the RGD motif, a significant increase in the efficiency of suicide gene therapy was demonstrated [13,14,15,34].

**Figure 8:**
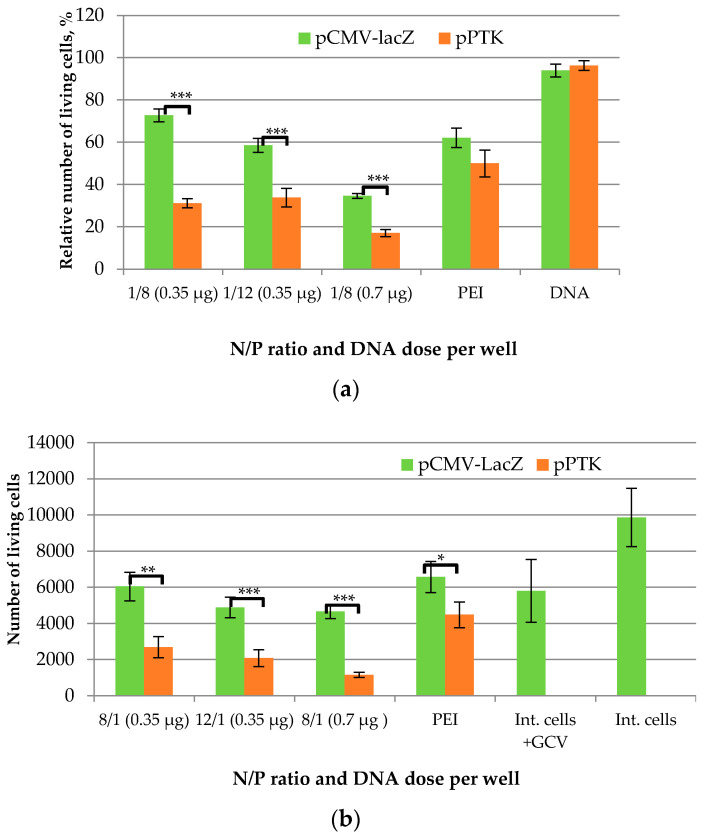
UF cell viability (**a**) and the number of living UF cells (**b**) after HSV thymidine kinase expression and GCV treatment as determined by Alamar Blue assay (**a**) and Trypan Blue exclusion assay (**b**). Values are the mean ± SEM of the mean of triplicates. * *p* < 0.05, ** *p* < 0.01, *** *p* < 0.001, compared to pCMV-LacZpolyplexes.

**Figure 9:**
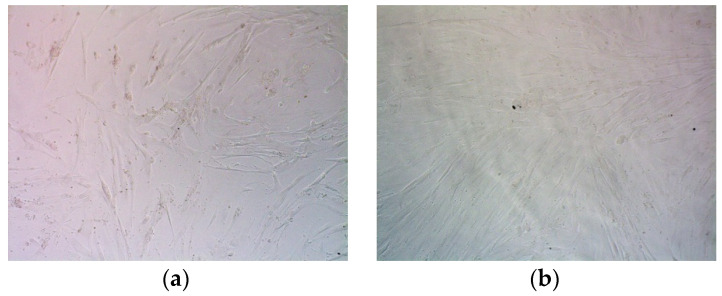

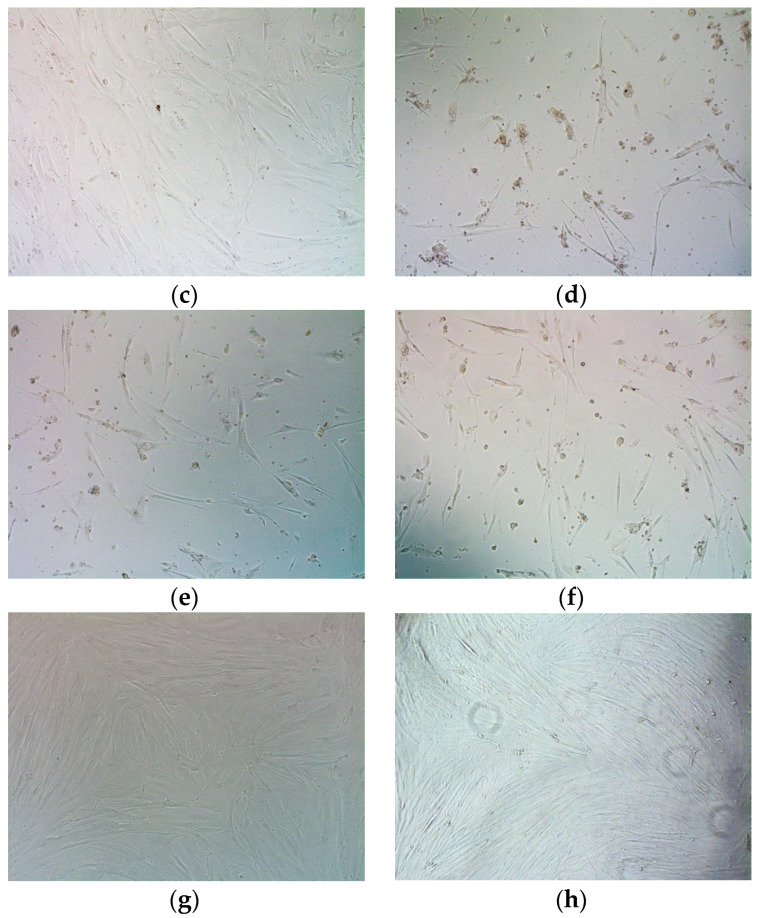
Representative microphotographs in bright field made 96 h after GCV treatment. The UF cells were transfected with R6p-cRGD/pCMV-lacZ polyplexes at N/Pratios of 8/1 (**a**,**b**) and 12/1 (**c**) with 0.7 µg (**a**) and 0.35 µg (**b**,**c**) of DNA per well; with R6p-cRGD/pPTK1 complexes at 8/1 (**d**,**e**) and 12/1 (**f**) charge ratios with 0.7 µg (**d**) and 0.35 µg (**e**,**f**) of DNA per well. Control wells contained GCV-treated intact cells (**g**) and untreated intact ones (**h**).

**Figure 10:**
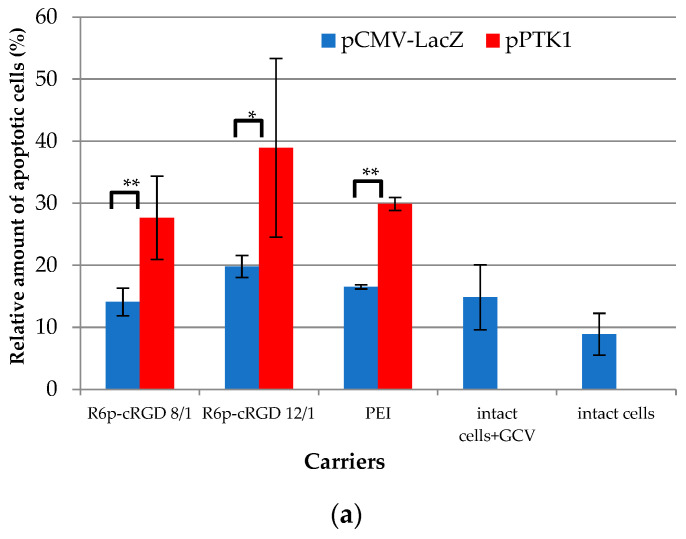

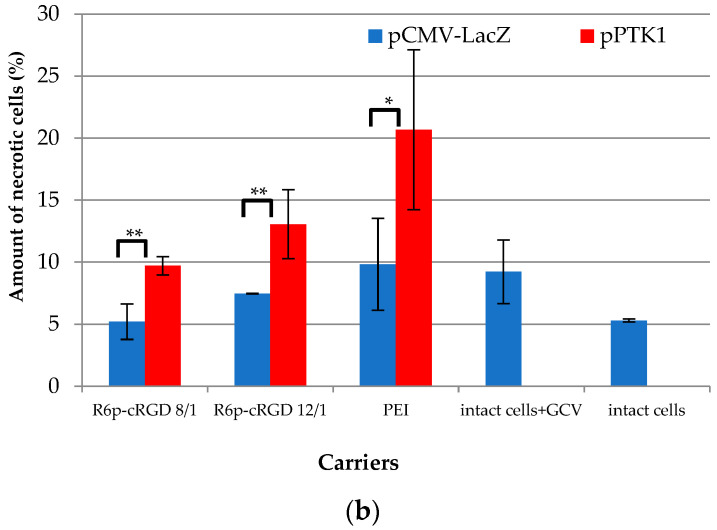
Relative amount of apoptotic (**a**) and necrotic (**b**) UF cells induced by GCV treatment after cell transfection with R6p-cRGD/DNA polyplexes formed with pPTK and pCMV-lacZ plasmids. Values are the mean ± SEM of the mean of four independent experiments. * *p* < 0.05, ** *p* < 0.01, compared to pCMV-lacZ-complexes.

**Figure 11:**
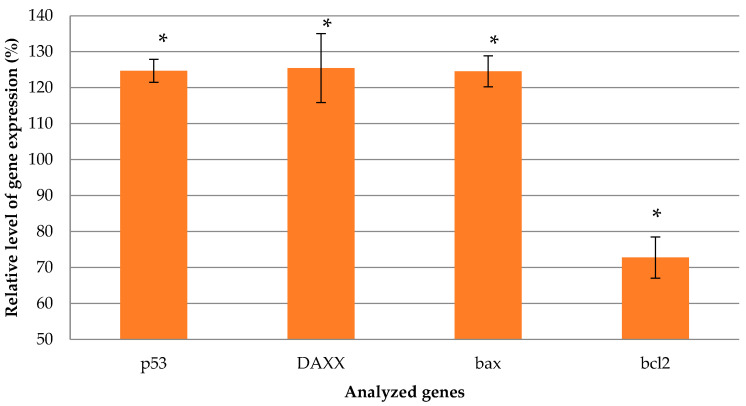
Relative gene expression levels of pro-apoptotic (p53, DAXX, bax) and anti-apoptotic factors in UF cells induced by GCV treatment after cell transfection with R6p-cRGD/DNA polyplexes formed with the pPTK plasmid. Values are the mean ± SEM of the mean of three independent experiments. * *p* < 0.05, compared to intact cells.

Herein, the suicide effect of R6p-cRGD/pPTK1 polyplexes at N/P ratios of 8/1 and 12/1 with 0.7 µg and 0.35 µg of DNA per well was evaluated in primary UF cells, obtained from patients after myomectomy. The cells were treated with GCV at a concentration of 50 µg /mL. Assessment of the suicide gene therapy effect included determination of cell proliferation efficiency using the AlamarBlue assay (Figure 8a) and the amount of living cells by the trypan blue exclusion method (Figure 8b). For a visual demonstration of the obtained results, we present micrographs of UF cells (Figure 9). Further, the evaluation of relative apoptotic and necrotic cell number by ApoDETECT annexin V-FITC kit (Figure 10), and pro- and anti-apoptotic factor expression analysis were carried out (Figure 11). The RGD1-R6/pCMV-lacZ polyplexes were used to prove suicide gene therapy effects rather than those associated with polyplex cytotoxicity. Intact cells and naked DNAs treated with GCV also served as controls.

An AlamarBlue assay conducted 4 days after GCV treatment demonstrated the effect of suicide gene therapy for R6p-cRGD/pPTK complexes (Figure 8a). Cell transfection with naked DNA, both pPTKandpCMV-lacZ, showed no decrease in cell viability in the presence of GCV. The therapeutic effect was observed in UF cells treated with GCV after R6p-cRGD/pPTK transfection at 8/1 and 12/1 charge ratios; we registered a 1.7–2.3-fold decrease in the cells’ proliferative activity compared to R6p-cRGD/pCMV-lacZ polyplexes. It should be noted that no suicide effect occurred when UF cells were transfected with PEI/pPTK1 complexes. The cytotoxicity caused by R6p-cRGD/pCMV-lacZ complexes with 0.35 µg of DNA did not exceed that of PEIpolyplexes. However, the DNA dose escalation resulted in an increase of polyplex cytotoxicity. Nevertheless, R6p-cRGD/pPTK polyplexes at an N/P ratio of 8/1 and a DNA dose of 0.35 µg were less toxic and showed high suicide efficiency at the level of 57% (Figure 8a). 

The Trypan Blue dye exclusion method showed a similar tendency in the assessment of the therapeutic effects (Figure 8b). No significant difference in the number of UF cells was observed upon cell transfection with pCMV-lacZ complexes and intact cells treated with GCV only. However, the number of viable UF cells transfected with R6p-cRGD/pPTK polyplexes decreased to 23–44% compared to R6p-cRGD/pCMV-lacZ complexes. Cell transfection with PEI/pPTK1 complexes resulted only in a 1.4-fold decrease in the population of viable UF cells. 

Accordingly, in micrographs, we demonstrate a significant decrease in the number of UF cells after transfection with R6p-cRGD/pPTK polyplexes and GCV treatment compared to controls (Figure 9).

The success of suicide gene therapy is largely associated with the “bystander effect”, when the phosphorylated GCV migrates to non-transfected cells through gap junctions or by endocytosis of apoptotic vesicles [60,61]. Activation of apoptosis, which was triggered by HSV-TK gene expression after UF cells transfection and subsequent treatment with GCV, was estimated with anApoDETECT annexin V-FITC kit (Figure 10a). Apoptosis is one of the best-studied forms of programmed cell death. Important signs of the apoptotic pathway are the exposition of phospholipid phosphatidylserine on the cell surface, which occurs at the initial or early stages of apoptosis, followed by membrane blebbing, nuclear fragmentation, a decrease in cell volume, and the formation of apoptotic bodies [62]. Annexin V is a protein that binds to phosphatidylserine, making it possible to detect the early apoptotic cells. The flow cytometry analysis after cell labeling with theApoDETECT annexin V-FITC kit showed that 28–39% of UF cells transfected by R6p-cRGD/pPTK polyplexes with 0.35 µg of DNA were annexin V-positive. This percentage was significantly different from that of R6p-cRGD/pCMV-lacZ complexes (14–20%), but detection of some annexin V-positive cells after the control polyplex transfection could be associated with adherent cell detachment via RGD-αvβ3 integrin interaction [63]. Staurosporine treatment showed the presence of approximately 50% of annexin V-positive UF cells (data not shown). UF cells transfection by control PEI/pPTK complexes also resulted in the appearance of cells at an early stage of apoptosis (29%), although these polyplexes did not induce a largedecrease in UF cells proliferative activity (Figure 8). 

After apoptosis, cells undergo programmed necrosis, which is accompanied by outer membrane disruption. Due to the membrane damage, propidium iodide can penetrate into the cells and be detected [64]. We found that 5% of intact cells were PI-positive (Figure 10b). UF cells transfection with R6p-cRGD/pCMV-lacZ polyplexes formed at 8/1 and 12/1 charge ratios resulted in 5.2% and 7.5% of PI-positive cells, respectively. After the cells’ treatment with R6p-cRGD/pPTK complexes, the percentage of necrotic cells was significantly higher and reached 9.7–13%. However, the percentage of necrotic UF cells was demonstrated to be lower than that of the apoptotic cells. The highest amount of necrotic cells (21%) was registered for PEI/pPTK complexes, whichis caused not only by suicide effects but mainly by the cytotoxicity of PEIpolyplexes [65]. 

The key point of suicide gene therapy is the termination of DNA replication which leads to apoptosis. Thus, we decided to evaluate whether there is a difference in the expression level of the apoptotic factor transcripts (p53, Bax, DAXX, Bcl-2). Herein, we demonstrated the increased level of transcription of pro-apoptotic factors (DAXX, Bax, p53) and a decrease in anti-apoptotic Bcl-2 transcripts, which is further evidence of successful TK gene delivery by the carrier and suicide gene therapy (Figure 11). p53 is one of the key molecules regulating apoptosis and is involved in both the extrinsic (deathreceptor-activating) and intrinsic (mitochondrial) pathways [66]. The regulating function of p53 is presented by transcription activation of a variety of apoptotic factors, such as pro-apoptotic Bcl-2 family proteins (such as Bax) and transcription suppression of anti-apoptotic Bcl-2 family proteins (Bcl-2) [67]. Death domain-associated protein (DAXX) is involved in the extrinsic pathway and its expression was increased after UF treatment in Eker rats by Ad-DNER vector [12]. The same work presented the difference in the protein level of Bcl-2 and Bax between Ad-DNER-treated and control UFs [12]. 

Taken together, our results indicate that UF cell incubation with R6p-cRGD/pPTK complexes stimulated cell death in a suicide-specific manner and that the cells were registered mostly at the early apoptosis stage rather than the necrosis stage. Moreover, the polyplex treatment resulted in a significant decrease in the proliferation activity of UF cells and the number of viable cells. So, the developed complexes are capable of targeted therapy and represent a promising delivery system for the successful application of suicide gene therapy of uterine leiomyoma.

## 3. Materials and Methods

### 3.1. Cell Lines

Human pancreatic (PANC-1) and human kidney (293T) cell lines were obtained from Cell Collection of the Institute of Cytology RAS (Saint Petersburg, Russia). The cells were maintained according to the standard method, “Fundamental Techniques in Cell Culture”, Sigma-Aldrich (Sailsbury, Wiltshire, UK). Primary UF cell lines were obtained previously after myomectomy in the D.O. Ott Research Institute of Obstetrics, Gynecology and Reproductology (Saint Petersburg, Russia) and maintained as described earlier [31,68]. 

### 3.2. Peptide Synthesis and Characterization

R6 (CHRRRRRRHC), cRGD(cyclic RGDyC(*Npys*)), and cyclo(RGDfK) peptides were synthesized with solid phase Boc-chemistry in NPF Verta, LLC (Saint Petersburg, Russia) and were stored as a dry powder at −20 °C (Table 1). The purity of the peptides was determined by high-performance liquid chromatography as 90–95%. The R6p carrier was obtained as described previously [30]. Briefly, the peptide was dissolved at a 30 mM concentration with 30% of DMSO, followed by oxidative polycondensation reaction for the next 96 h. In the case of R6p-cRGD, the cRGD moiety dissolved at 30 mM with 30% of DMSO added to R6p before polycondensation at the ratio of 1 cRGD molecule to 2 R6 molecules. The resultant R6p and R6p-cRGD were stored dissolved in water, 2 mg/mL at −70 °C. The relative number of free thiol groups was estimated by Ellman’s assay and subsequently calculated as (P/Pf) × 100%, where Pf is the absorbance of unpolymerized peptide R6 [30]. The carrier molecular weight was analyzed on an AB Sciex 5800 TOF/TOF tandem time-of-flight mass spectrometer (AB Sciex, Foster City, CA, USA) in a linear mode; sinapic acid served as a matrix substance (Tables S1 and S2).

### 3.3. Reporter Plasmids 

The pCMV-lacZ plasmid with the β-galactosidase gene was gifted from Prof. B. Sholte, Erasmus University Rotterdam, Netherlands. The pEXPR-IBA5-eGFP plasmid with green fluorescence protein (GFP) gene was obtained from IBA GmbH, Göttingen, Germany. The pPTK1 plasmid containing the HSV1 herpes virus thymidine kinase gene was provided by Dr. S.V. Orlov from the Institute of Experimental Medicine, Saint Petersburg, Russia. The plasmids were isolated according to the standard alkaline lysis technique [69].

### 3.4. Complex Preparation, DNA-Binding and Protection Assays, DS and DTT Treatment

DNA/peptide complexes were prepared at various N/P ratios (peptide nitrogen/DNA phosphorus ratio), as described previously [30,31]. The plasmid DNA was diluted to 20 g/mL in Hepes-buffered mannitol (HBM) (5% (*w*/*v*) mannitol, 5 mM Hepes, pH 7.5) and then anequal volume of peptides at the required charge ratio in HBM was added to the DNA solution, followed by vortexing. Complexes were incubated at room temperature for 30 min. Polyethyleneimine (branched PEI 25 kDa; Sigma-Aldrich, St. Louis, MO, USA) was used as 0.9 mg/mL (pH 7.5) aqueous stock solution, stored at +4 °C. The ratio of PEI to DNA was 8/1.

Peptide binding to DNA was studied using the ethidium bromide (EtBr) fluorescence quenching method at different nitrogen-to-phosphate ratios (0.1–5) [70]. Fluorescence measurements were performed in a Wallac 1420D scanning multilabel counter (PerkinElmer Wallac Oy, Turku, Finland) in emission fluorescence at 590 nm (544 nm excitation). EtBr displacement was calculated as (F − Ff)/(Fb − Ff), where Ff and Fb are the fluorescence intensities of EtBr in the absence and presence of DNA, respectively.

DNAse I protection assay was carried out for complexes formed at a 0.1–5 N/P ratio when adding to them 0.5 units of DNase I (Ambion, Austin, TX, USA) for 30 min at 37 °C with 2 min subsequently of DNAse I activation. Then, 0.1% trypsin was added for DNA overnight release at 37 °C and the DNA integrity was analyzed in 1% agarose gel [71].

Dextransulfate (DS)(Sigma–Aldrich, St. Louis, MO, USA) was added to the complexes at three-fold charge excess relative to the carrier for the next 24 h of incubation and analyzed by EtBrexclusion assay. 

The dithiotreitol(DTT) at 200 mM was incubated with the complexes for 1 h at 37 °C and analyzed by SYBR-Green exclusion assay in a Wallac 1420D at an emission fluorescence of590 nm (585 nm excitation). SybrGreen displacement was calculated similarly to the EtBr assay [34].

### 3.5. Measurement of Size and ʐ-Potential of Peptide/DNA Complexes 

The peptide/DNA complexes were prepared as described above in quantities of 5 µg of DNA per sample. The size of the complexes was determined using dynamic light scattering, and the zeta potential was determined by microelectrophoresis. Three independent measurements were performed on a zetasizer NANO ZS (Malvern Instruments, Malvern, UK).

### 3.6. Gene Transfer 

PANC-1 cells were seeded at a density of 5.0 × 10^4^ cells per well in 48-well plates and incubated overnight. Before transfection, the cell culture medium was replaced with serum-free medium. DNA complexes, prepared as above (2 µg of DNA in each well), were added and incubated with cells for 4 h. Then, the transfection medium was replaced with FBS-containing medium and the cells were incubated for the next 48 h. 

The β-galactosidase activity in cell extracts was measured with methyl-umbelliferyl-β-D-galactopyranoside (MUG) and normalized by the total protein concentration, measured with Bradford reagent (Helicon, Moscow, Russia), as described previously [30]. 

For the competition transfection study a 10-fold excess of cyclo(RGDfK) peptide was added to the cells 15 min before complex treatment, followed by the procedures described above. 

GFP expression was determined by flow cytometry using a BD FACS-Canto II cytofluorimeter. Transfection efficacy was evaluated as a percentage of GFP-positive cells. 

### 3.7. Cellular Uptake of Peptide/DNA Complexes 

PANC-1 or 293T cells were seeded at a density of 5 × 10^4^ cells/well in 48-well plates a day before the experiments. Peptide/DNA complexes were formed with added YOYO-1 iodide based on 1 molecule of the dye per 50 base pairs. Transfection was performed according to the protocol described above. After 2 h of incubation with complexes, the cells were washed three times in 1× PBS (pH 7.2) and once with 1M NaCl (in 1× PBS). The cells were detached and incubated with propidium iodide solution, as described previously [34]. Then, the living cells, at a rate of 10,000 per sample, were analyzed by flow cytometry with a BD FACS-Canto II cytofluorimeter. The results were presented as RFU/cell. 

### 3.8. Cytotoxicity Assay 

The cytotoxicity of DNA/peptide complexes was evaluated in PANC-1 cells using AlamarBlue assay (BioSources International, San Diego, CA, USA), as described previously [71]. Carrier/DNA complexes were prepared at the rate of 0.7 µg of DNA per well of a 96-well plate. Fluorescence measurements were performed in a Wallac 1420D scanning multilabel counter in emission fluorescence at 590 nm (544 nm excitation). The relative fluorescence intensity was counted as (F − Ff)/(Fb − Ff) × 100%, where Fb and Ff are the fluorescence intensities in untreated controls and without cells, respectively.

### 3.9. Suicide Gene Therapy

The primary UF cells were seeded on 96-well plates at 1.5 × 10^4^ cells per well for overnight incubation. Transfections were performed in serum-free medium with 0.7 µg and 0.35 µg of DNA (pPTK1 or pCMV-lacZ plasmid) per well. After 2 h of cell incubation with complexes, the medium was replaced with FBS-containing medium and the cells were incubated for the next 24 h. Further, the medium was replaced withfresh mediumbut with ganciclovir at a concentration of 50 μg/mL and the cells were allowed to grow for 96 or 24 h [34]. 

After 96 h of incubation, the medium was replaced withthe same one but with 10% Alamar Blue solution added, and the cells were incubated for the next 2 h. Fluorescence measurements were performed in a Wallac 1420D scanning multilabel counter in emission fluorescence at 590 nm (544 nm excitation). The cell proliferation activity was estimated by the number of living cells, calculated as (F − Ff)/(Fb − Ff), where Fb and Ff are the fluorescence intensities in untreated controls and without cells, respectively. Micrographs of the cells were taken at 100× magnification with a AxioObserver Z1 microscope (Carl Zeiss, Oberkochen, Germany) equipped with the AxioVision program.

Trypan Blue dye was used to count the number of living cells after 96 h of incubation. UF cells were harvested with 0.25% Trypsin-EDTA (Thermo Fisher Scientific, Carlsbad, CA, USA), followed by addition of 0.4% Trypan Blue solution (Sigma-Aldrich, Munich, Germany) at a 1:1 volume ratio for 15 min. The Trypan Blue-negative cells were counted with a hemocytometer (MiniMedProm, Dyatkovo, Russia).

The relative number of apoptotic and necrotic cells was determined after 24 h of incubation with the ApoDETECT annexin V-FITC kit (Invitrogen, Darmstadt, Germany), according to the manufacturer’s recommendations. The cells were detached, treated with the kit and analyzed with aBD FACS-Canto II cytofluorimeter.

For the gene expression analysis, total RNA extraction and quantitative real-time PCR analysis were performed as previously described [72]. The following primers were used: Bax forward primer 5′-TTC TGA CGG CAA CTT CAA CTG G-3′, reverse primer 5′-AGG AAG TCC AAT GTC CAG CC-3′ [73]; p53 forward primer 5′-TAA CAG TTC CTG CAT GGG CGG C-3′, reverse primer 5′-AGG ACA GGC ACA AAC ACG CAC C-3′ [74];DAXX forward primer 5′-CTG AAA TCC CCA CCA CTT CC-3′, reverse primer 5′-CTG AGCAG CTG CTT CAT CTT C-3′ [75]; Bcl-2 forward primer 5′-GAG GAT TGT GGC CTT CTT TG-3′, reverse primer 5′-GCC GGT TCA GGT ACT CAG TC-3′ [76]; and the endogenous reference gene GAPDH was detected using forward 5′-CGC CAG CCG AGC CAC ATC-3′, reverse primer 5′-CGC CCA ATA CGA CCA AAT CCG-3′. The samples were measured three times and a final result was inferred by averaging the data. The values are presented as mean ± SEM of the means obtained from two independent experiments.

### 3.10. Statistical Analysis

Statistically significant differences were obtained with the Mann–Whitney Utest and the Student’s *t*-test using Instat 3.0 (GraphPad Software Inc., San Diego, CA, USA). *p* < 0.05 was considered statistically significant.

## 4. Conclusions

In the current study, we developed a promising non-viral gene delivery system based on cysteine-flanked arginine-rich peptides. The developed carrier, R6p-cRGD, was modified with a cyclic RGD ligand during a polycondensation reaction, as was proved by massspectrometry. Physicochemical experiments reveal that R6p-cRGD can form small-sized stable complexes with DNA thatprotect it from nuclease degradation. Cell transfection experiments confirmed the important role of the ligand modification for the specificity of DNA delivery to αvβ3 integrin-expressing cells. Complexes of the developed carrier and HSV-1 thymidine kinase-encoding plasmid were extensively studied in model experiments on suicide gene therapy of uterine leiomyoma in vitro. We showed that R6p-cRGD-mediated HSV-1 thymidine kinase gene expression in uterine leiomyoma cells reduced their proliferative activity and increased the number of apoptotic and necrotic cells. These findings were confirmed by the increased expression of pro-apoptotic factors and a decreased expression of anti-apoptotic factor Bcl-2 in uterine leiomyoma cells after R6p-cRGD-mediated transfection. Thus, we can conclude that the developed R6p-cRGD carrier can be used for further efforts in the development of uterine leiomyoma suicide gene therapy.

## Figures and Tables

**Table 1:** Design and composition of the synthesized peptides and peptide carriers.

	Name	Composition
	R6	CHRRRRRRHC
Monomers	cRGD	C(*Npys*)RGDy **|___________|**
Carriers	R6p R6p-cRGD	(CHRRRRRRHC)_n_ cRGD-(CHRRRRRRHC)_n_-cRGD

**Table 2:** Size and ʐ-potential of the carrier/DNA complexes.

Carrier	N/P Ratio	Size (nm) ± SD	ʐ -Potential (mV) ± SD
R6p	8/1	120.9 ± 0.17	35.6 ± 0.21
R6p	12/1	110.3 ± 9.12	35.5 ± 0.81
R6p-cRGD	8/1	104.5 ± 0.15	32.0 ± 1.17
R6p-cRGD	12/1	178.4 ± 24.32	31.5 ± 0.61

## Data Availability

The data presented in this study are available on request from the corresponding author. The data are not publicly available due to restrictions of the subjects’ agreement.

## Supplementary Materials

The following are available online at https://www.mdpi.com/article/10.3390/ijms23031164/s1.
