# Supplementary material for: Polycondensed Peptide Carriers Modified with Cyclic RGD Ligand for Targeted Suicide Gene Delivery to Uterine Fibroid Cells
Source: Int J Mol Sci. 2022 Jan 21;23(3):1164. doi: 10.3390/ijms23031164 (PMC8835468; doi:10.3390/ijms23031164)
Supplement: Supplementary file 1 [file ijms-23-01164-s001.zip › ijms-1558567-supplementary.pdf]

*Supporting Information for the Article*

# Polycondensed peptide carriers modified with cyclic RGD ligand for targeted suicide gene delivery to uterine fibroid cells

**Anna Egorova[1], Sofia Shtykalova[1], Marianna Maretina[1], Natalia Shved[1], Alexander Selutin[2], Dmitriy Deviatkin[1], Sergey Selkov[2], Vladislav Baranov[1] and Anton Kiselev[1,*]**

[1] Department of Genomic Medicine, D.O. Ott Research Institute of Obstetrics, Gynecology and Reproductology, Mendeleevskaya Line 3, 199034 Saint-Petersburg, Russia; A.E. egorova_anna@yahoo.com; S.Sh. sofia.shtykalova@gmail.com; M.M. marianna0204@gmail.com; N.S. natashved@mail.ru; D.D. dimi02121@gmail.com; V.S. baranov@vb2475.spb.edu; A.K. ankiselev@yahoo.co.uk

[2] Department of Immunology and Intercellular Interactions, D.O. Ott Research Institute of Obstetrics, Gynecology and Reproductology, Mendeleevskaya Line 3, 199034 Saint-Petersburg, Russia; A.S. a_selutin@yahoo.com; S.S. selkovsa@mail.ru

* Correspondence: ankiselev@yahoo.co.uk

**Contents**

**Table S1.** Molecular weight of the R6p carrier measured using MALDI-TOF mass-spectrometry……………........................................................................................................3

**Table S2.** Molecular weight of the R6p-cRGD carrier measured using MALDI-TOF mass-spectrometry………..................................................................................................4

**Table S1.** Molecular weight of the R6p carrier measured using MALDI-TOF mass-spectrometry. n.d. - not detected.

| **Molecular weight, Da** | | **Polymerization rate** |
|---|---|---|
| **theoretical** | **experimental** | |
| 2871.44 | 2867.3 | $(R6)_2$ |
| 4307.16 | 4298.82 | $(R6)_3$ |
| 5742.88 | 5732.63 | $(R6)_4$ |
| 7178.6 | 7160.65 | $(R6)_5$ |
| 8614.32 | 8871.83 | $(R6)_6$ |
| 10050.04 | 9485.35 | (R6)7 |
| 11485.76 | 11330.87 | $(R6)_8$ |
| 12921.48 | n.d. | $(R6)_9$ |
| 14357.2 | 14809.73 | $(R6)_{10}$ |
| 15792.92 | 15986.75 | $(R6)_{11}$ |
| 17228.64 | 17329.89 | $(R6)_{12}$ |
| 18664.36 | 19056.81 | $(R6)_{13}$ |
| 20100.08 | 20447.93 | $(R6)_{14}$ |
| 21535.8 | n.d. | $(R6)_{15}$ |
| 22971.52 | 22301.45 | $(R6)_{16}$ |
| 24407.24 | 24514.12 | $(R6)_{17}$ |
| 25842.96 | 25673.97 | $(R6)_{18}$ |
| 27278.68 | 27427.64 | $(R6)_{19}$ |

**Table S2.** Molecular weight of the R6p-cRGD carrier measured using MALDI-TOF mass-spectrometry.

| Molecular weight, Da | | Polymerization rate |
|---|---|---|
| **theoretical** | **experimental** | |
| 2184.51 | 2181.07 | (R6)-cRGD |
| 2933.3 | 2866.63 | cRGD-(R6)-cRGD |
| 2871.44 | 2871.28 | $(R6)_2$ |
| 3620.23 | 3460.36 | $(R6)_2$-cRGD |
| 7927.39 | 7387.04 | $(R6)_5$-cRGD |
| 8614.32 | 8480.05 | $(R6)_6$ |
| 8676.18 | 8676.99 | cRGD-$(R6)_5$-cRGD |
| 9363.11 | 9719.7 | $(R6)_6$-cRGD |
| 10111.9 | 9883.27 | cRGD-$(R6)_6$-cRGD |
| 10798.83 | 10572.81 | $(R6)_7$-cRGD |
| 12234.55 | 12394.15 | $(R6)_8$-cRGD |
| 12983.34 | 13354.83 | cRGD-$(R6)_8$-cRGD |
| 14419.06 | 14398.1 | cRGD-$(R6)_9$-cRGD |
| 15854.78 | 16001.11 | cRGD-$(R6)_{10}$-cRGD |
| 16541.71 | 16323.56 | $(R6)_{11}$-cRGD |
| 17290.5 | 17383.41 | cRGD-$(R6)_{11}$-cRGD |
| 17977.43 | 17383.41 | $(R6)_{12}$-cRGD |
| 19413.15 | 19499.59 | $(R6)_{13}$-cRGD |
| 20161.94 | 20563.55 | cRGD-$(R6)_{13}$-cRGD |
| 21535.8 | 21402.16 | $(R6)_{15}$ |
| 21597.66 | 21954.36 | cRGD-$(R6)_{14}$-cRGD |
| 22284.59 | 22167.61 | $(R6)_{15}$-cRGD |
| 23720.31 | 23628.45 | $(R6)_{16}$-cRGD |
| 25156.03 | 25471.4 | $(R6)_{17}$-cRGD |
| 25904.82 | 25864.05 | cRGD-$(R6)_{17}$-cRGD |
| 26591.75 | 26602.05 | $(R6)_{18}$-cRGD |
| 27340.54 | 27487.68 | cRGD-$(R6)_{18}$-cRGD |
